# A causal meta-analysis framework for clinical trials with unequal randomization ratios

**DOI:** 10.1017/rsm.2025.10069

**Published:** 2026-03-05

**Authors:** Dazheng Zhang, Bingyu Zhang, Lu Li, Haitao Chu, Yong Chen

**Affiliations:** 1 The Center for Health AI and Synthesis of Evidence (CHASE), University of Pennsylvania, Philadelphia, PA, USA; 2 Department of Biostatistics, Epidemiology and Informatics, Perelman School of Medicine, The University of Pennsylvania, Philadelphia, PA, USA; 3 The Graduate Group in Applied Mathematics and Computational Science, School of Arts and Sciences, University of Pennsylvania, Philadelphia, PA, USA; 4 Statistical Research and Data Science Center, Pfizer Inc., USA; 5 Division of Biostatistics and Health Data Sciences, University of Minnesota Twin Cities, Minneapolis, MN, USA; 6 Leonard Davis Institute of Health Economics, Philadelphia, PA, USA; 7 Penn Medicine Center for Evidence-based Practice (CEP), Philadelphia, PA, USA; 8 Penn Institute for Biomedical Informatics (IBI), Philadelphia, PA, USA

**Keywords:** causal estimands, clinical trials, meta-analysis, the average treatment effect

## Abstract

Meta-analysis synthesizes evidence from multiple randomized clinical trials and informs evidence-based practices across various medical domains. Recently, causally interpretable meta-analysis has been proposed and applied to treatment evaluations for target populations, requiring individual participant data (IPD). Standard meta-analysis assumes transportability or exchangeability of a (conditional) relative effect (such as relative risk or odds ratio), which may be violated when the relative effects are correlated with the baseline risks across clinical trials. In addition, the weighted average of some study-specific effect measures such as the (log) odds ratios or the (log) hazard ratios is non-collapsible and does not correspond to any target population. Furthermore, when the randomization ratios between treated versus untreated arms vary across trials, confounding bias may occur. To address these challenges, we propose a causal meta-analysis (CMA) framework using only aggregated data, enabling causally interpretable and accurate estimation for different target populations. The CMA adjusts its weights for treatment effect across various target populations, including the average treatment effect (ATE), the ATE on the treated (ATT) population, the ATE on the control (ATC) population, and the ATE in the overlap (ATO) population. Mathematically, we discover the connection between traditional meta-analysis estimators and CMAs. For example, the Mantel–Haenszel weighted meta-analysis is equivalent to the CMA with ATO.

## Highlights

**What is already known?**

- Traditional meta-analysis synthesizes data from multiple clinical trials to inform treatment evaluation but assumes exchangeability or transportability of effect measures, which may be violated when baseline risks vary across studies.
- Causally interpretable meta-analysis methods require to use individual patient data to conduct evidence synthesis for target populations.

**What is new?**

- The proposed causal meta-analysis (CMA) framework estimates treatment effects for target populations using aggregated data, adjusting for confounding due to varied randomization ratios.
- The CMA framework connects traditional meta-analysis estimators with causal estimands like average treatment effect (ATE), ATE on the treated (ATT) population, ATE on the control (ATC) population, and ATE in the overlap (ATO) population, broadening interpretability and precision.

**Potential impact for RSM readers outside the authors’ field:**

- The CMA framework’s ability to estimate causally interpretable treatment effects across diverse populations enhances the applicability of meta-analytic results across clinical and methodological disciplines.
- This approach provides a practical tool for researchers needing reliable causal estimates from aggregate data across clinical trials.

## 1 Introduction

Meta-analysis is an essential methodology in medical research, offering a systematic approach for combining and contrasting data from multiple clinical studies.^1^^–^ ^3^ This technique consolidates disparate findings into a coherent understanding, recognized as a best practice at the pinnacle of the evidence hierarchy.^4^ It has been extensively utilized in areas such as diagnostic accuracy, drug safety, health economics, and genetic studies.^4^^–^ ^14^ This wide-ranging utilization underscores its critical role in enhancing evidence-based practices and advancing patient care.

Despite the numerous advantages of meta-analysis and its extensive applications, how to best assign weights to different studies in a meta-analysis remains a crucial question. In the common-effect meta-analysis, a well-accepted recommendation is to use the inverse variance of each study, as it possesses the statistical properties of unbiasedness and minimum variance.^15^ However, in instances where the variance of the estimate from each study is unstable due to the small sample sizes, an alternative approach involves weighting by the study sample size.^16^ This alternative method has been shown to achieve a smaller mean-square error compared to the inverse variance weighting approach^17^ and to provide better interpretation. Another viable approach is to use weights based on the geometric mean of the number of subjects in the treated and control groups within each study, known as the Mantel–Haenszel method.^18^^,^ ^19^ This method proves particularly applicable and beneficial when dealing with rare event rates for studies, especially when some studies have zero or a small number of events.^15^

A discrepancy in treatment evaluation decisions has arisen within meta-analyses. The treatment effect, inherently tied to causal inference on the target population, typically diverges from the traditional meta-analysis method. A recently developed meta-analysis approach aims to provide a causal interpretation of the treatment effect, thereby enhancing decision-making in treatment evaluation for the target population.^20^^–^ ^23^ Rott et al. compared traditional (fixed-effect and random-effect) meta-analysis estimators with newly developed causally interpretable counterparts in empirical experiments and real-world studies.^24^ This study found that traditional meta-analysis methods can perform comparably to causally interpretable approaches, particularly in settings where covariates do not meaningfully modify treatment effects.

The newly developed causally interpretable meta-analysis method requires access to individual participant data (IPD), which is often impractical. In contrast, standard fixed-effect (or common-effect) meta-analysis can synthesize aggregated data by weighting study-specific relative effects using inverse variance weights. However, when non-collapsible effect measures such as odds ratios or hazard ratios are used,^25^^–^ ^28^ the overall weighted relative effect does not correspond to the average treatment effect (ATE) in any identifiable target population. Consequently, evaluations of any specific treatment effects for a particular target population from this approach become problematic. Moreover, this method assumes the transportability or exchangeability of conditional relative effects; for example, study-specific relative risk or odds ratios are exchangeable. These assumptions may not hold when baseline event rates differ substantially across studies and relative effects are associated with baseline event rates. According to a recent meta-epidemiologic study, approximately 28% of meta-analyses demonstrate a significant association between the treatment effect and control group event rate.^29^ Furthermore, the sample size ratios between treated and untreated groups may vary across different studies. It may introduce confounding bias, thereby complicating the estimation procedure and affecting the overall accuracy of the results.

In this study, we aim to estimate the treatment effect on different target populations while addressing the between-study confounding effect using only aggregated data. Specifically, we develop a causal meta-analysis (CMA) estimand framework focusing on various populations: the overall population in both the treatment and control groups, the treated group alone, the control group alone, and the overlap population across studies. The overlap population includes patients with the most similar characteristics between the comparison groups, aligning with the concept of clinical equipoise.^30^^,^ ^31^ These target populations are considered as different weighted populations across studies, with varying weights designed to target different estimands. The estimands for these weighted populations fall into a category of causal estimands known as weighted ATE (WATE).^32^ Several causal estimands are included within WATE, including the ATE, the ATE on the treated (ATT) population, the ATE on the control (ATC) population, and the ATE in the overlap (ATO) population.

To estimate the WATE and eliminate the between-study confounding effects, we propose a CMA framework under the fixed-effect meta-analysis.^33^ We provide the estimators for each target estimand derived from the CMA estimator. We theoretically establish equivalence between the CMA estimator and the propensity score weighting estimator for WATE, assuming between-study heterogeneity and no confounders within each study (due to randomization). Subsequent sections demonstrate connections between the CMA estimator and traditional fixed-effect meta-analysis estimators, particularly when the target estimand is ATE or ATO. To show the difference between the CMA estimators for different estimands, we generated a synthesized dataset and conducted the CMA estimators. To the best of our knowledge, our proposed method is the first to evaluate treatments for target populations using only aggregated data while accounting for potential confounding bias. Additionally, this is the first study to connect the traditional meta-analysis with target populations, marking a significant advancement in the field.

## 2 Notation and methods

We consider K randomized trials to estimate treatment effects for target populations, assuming treatments are time-fixed. Each estimand corresponds to a distinct target population, defined by specific combinations of the populations enrolled in the included trials, rather than by a hypothetical or external population. We denote the treatment indicator by A , which has two values, 0 if the treatment is not given and 1 if the treatment is given. We denote two potential outcomes Y(0) and Y(1) corresponding to the outcome if the treatment is given (A=1) or the treatment is not given (A=0) . Furthermore, we assume that there are in total n subjects within K trials. We denote the study indicator, representing which study a subject belongs to, as X . For each subject, we observed the outcome denoted by $Y_{ij}$ after the randomization of the treatment within each study. The value of $Y_{ij}$ can be 0 or 1, where 1 means the subject has the event and 0 means the subject does not have the event. Let $n_{aj}$ and ${\hat{p}}_{aj}$ be the number of subjects and estimated event rate for the j th study (j=1,…,K) in the treatment arm (A=1) or control arm (A=0) . Let $n_{j}=\sum_{a=1}^{2}n_{aj}$ be the study size for the x th study (j=1,…,K) . We made the following assumptions to make the potential outcomes Y(1),Y(0) identifiable.

### 2.1 Assumptions

Assumption 1 **consistency of potential outcomes**

For the i th participant within the j th trial, given the treatment assignment $A_{ij}=a$ , the observed outcome satisfies $Y_{ij}(a)=Y_{ij}$ , for a=0,1 .

This assumption implies that the observed outcome corresponds exactly to the potential outcome under the treatment actually received. In the context of our framework, Assumption 1 further requires that there is no direct effect of the study indicator X (i.e., study membership) on the potential outcomes, conditional on treatment assignment. In other words, once treatment is assigned, the specific study from which an individual originates does not modify the causal effect of the treatment on the outcome. This assumption also rules out treatment variation irrelevance (i.e., no different versions of the treatment within levels of A ) and assumes no interference between units (stable unit treatment value assumption [SUTVA]), such that one participant’s outcome is unaffected by the treatment assignment of other participants.^34^

Assumption 2 **no unmeasured confounding variables**

The potential outcomes are independent of treatment assignment conditional on study membership: $Y_{ij}(0),Y_{ij}(1)⊥A_{ij}|X_{ij}$ .

Assumption 2 implies that the study indicator $X_{ij}$ is the sole confounding variable relevant to the treatment assignment in the pooled analysis. This assumption is reasonable in the context of meta-analysis of randomized clinical trials. While randomization within each trial ensures that pretreatment covariates are balanced between treatment arms, differences across studies (captured by $X_{ij}$ ) may induce confounding in the pooled analysis when data from multiple studies are combined. Thus, conditioning on study membership is essential to account for such between-study heterogeneity in the pooled analysis.

**Figure 1:**
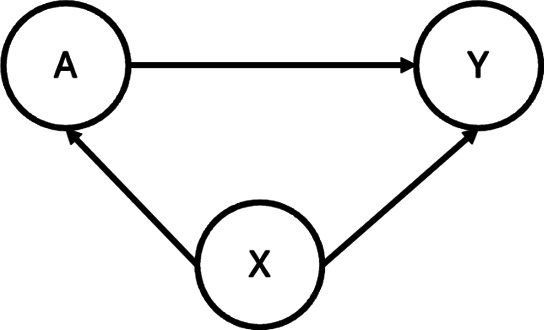
The directed acyclic graph (DAG) illustrating the assumed causal relationships between the treatment (A), outcome (Y), and a study indicator (X). In this structure, X confounds the relationship between A and Y, as it influences both variables.

As demonstrated in Figure 1, heterogeneity in study design can induce confounding in pooled analyses of randomized trials. Specifically, when different studies employ varying randomization ratios, the study indicator $X_{ij}$ may be associated with treatment assignment $A_{ij}$ (i.e., $X_{ij}\to A_{ij}$ ). In addition, heterogeneity in baseline event rates across studies—for example, due to differences in patient characteristics or clinical settings—may induce an association between $X_{ij}$ and the observed outcome $Y_{ij}$ (i.e., $X_{ij}\to Y_{ij}$ ). Thus, $X_{ij}$ serves as a between-study confounder that must be accounted for to obtain unbiased causal effect estimates. Importantly, within each individual study, randomization ensures that no other unmeasured pretreatment confounders exist. Therefore, we posit that conditioning on $X_{ij}$ is sufficient to control for all confounding between treatment assignment and outcome in the combined analysis.^34^ When different studies have different randomization ratios, $X_{ij}$ , the study indicator is associated with the treatment assignment. $X_{ij}$ may also be related to the observed outcome $Y_{ij}$ . This is because the prevalence of $Y_{ij}$ might be associated with the heterogeneous baseline event rates across studies. Thus, $X_{ij}$ serves as a between-study confounding variable. However, within each study, all other pretreatment confounders are controlled because of the randomization procedure. Thus, we can assume there is only a covariate $X_{ij}$ serves as a confounder for the potential outcomes.

Assumption 3 **treatment positivity**

For each treatment ( a=0,1 ), the probability of receiving that treatment, conditional on study membership, is strictly positive: P(A=a|X)>0 .

This assumption holds because, within each study, treatment assignment follows a randomization scheme. Therefore, the probability of receiving either treatment is strictly positive and bounded away from zero and one, thereby guaranteeing sufficient overlap in treatment assignment across studies.

### 2.2 Proposed Causal Meta-Analysis Framework Under Assumptions

To adjust the confounder effect of $X_{ij}$ , propensity score weighting is a commonly used approach in the causal inference literature.^35^^,^ ^36^ It creates a pseudo-weighted population where the covariate distribution in the treated subjects is the same as the covariate distribution in the not-treated subjects. The causal estimand $\tau_{w}$ for the weighted population is displayed as:

$$\begin{array}{r} \tau_{w}=\frac{E\left(h(X)\left(\mu_{1}(X)-\mu_{0}(X)\right)\right)}{E\left(h(X)\right)}, \end{array}$$

where $\mu_{1}(X)=E\left(Y(1)|X\right),\mu_{0}(X)=E\left(Y(0)|X\right),$ and h(X) is the weighting function of the propensity score e(X) to shift the population from its original covariate distribution f(x) . With different forms of h(X) , the WATE can be equivalent to different target estimands, such as the ATE when h(X)=1 .

The individual estimated weight for the i th subject within the j th study, denoted by $m_{ij}$ , is a function of the estimated propensity score ${\hat{e}}_{ij}=\hat{P}\left(A_{ij}=1|X_{ij}\right)$ from logistic regression. When the form of h(X) is a function of the propensity score, we have $\hat{h}\left(X_{ij}\right)=h\left({\hat{e}}_{ij}\right)$ . In the treatment arm, $m_{ij}$ is equal to $\frac{\hat{h}\left(X_{ij}\right)}{{\hat{e}}_{ij}}$ . In the control arm, it is equal to $\frac{\hat{h}\left(X_{ij}\right)}{1-{\hat{e}}_{ij}}$ . With the weights $m_{ij}$ , the following estimator estimates the WATE, that is,

(1) $$\begin{array}{r} {\hat{\tau }}_{w}=\frac{\sum_{j=1}^{K}\sum_{i=1}^{n_{j}}A_{ij}Y_{ij}m_{ij}}{\sum_{j=1}^{K}\sum_{i=1}^{n_{j}}A_{ij}m_{ij}}-\frac{\sum_{j=1}^{K}\sum_{i=1}^{n_{j}}\left(1-A_{ij}\right)Y_{ij}m_{ij}}{\sum_{j=1}^{K}\sum_{i=1}^{n_{j}}\left(1-A_{ij}\right)m_{ij}}.\; \end{array}$$

Under the assumption for the between-study confounders and no within-study confounders, the estimated propensity scores for all subjects within the j th study are the same because $X_{ij}$ is a study indicator. Moreover, the estimate of the propensity score is thus reduced to the estimated randomization ratio for the j th study. In other words, for the i th subject,

(2) $$\begin{array}{r} {\hat{e}}_{ij}={\hat{\lambda }}_{j}I\left(X_{ij}=j\right)\;j=1,..\;,K,\; \end{array}$$

where ${\hat{\lambda }}_{j}=\frac{n_{1j}}{n_{j}}$ is the proportion of treatments in the j th study and I(⋅) is an indicator function. Thus, $\hat{h}\left(X_{ij}\right)={\hat{h}}_{j}I\left(X_{ij}=j\right)$ with ${\hat{h}}_{j}=h\left({\hat{\lambda }}_{j}\right)$ when j=1,…,K . Based on the aggregated data by using Equation (2) and the corresponding ${\hat{h}}_{j}$ , we propose the fixed-effect CMA estimator:

(3) $$\begin{array}{r} {\hat{\tau }}_{CMA}=\sum_{j=1}^{K}w_{j}\left({\hat{p}}_{1j}-{\hat{p}}_{0j}\right),\; \end{array}$$

where $w_{j}={\left(\sum_{j=1}^{K}n_{j}{\hat{h}}_{j}\right)}^{-1}n_{j}{\hat{h}}_{j}$ . In Supplementary Materials, we show how the CMA estimator shown in Equation (3) can be obtained from Equation (1) by only using the aggregated data ${\hat{h}}_{j}$ .

The variance of the ${\hat{\tau }}_{CMA}$ can be calculated by finding the influence function of $\tau_{w}$ . The variance estimator can thus be obtained by ${\left(n^{2}\;{\hat{\theta }}^{2}\right)}^{-1}\sum_{j=1}^{K}{\hat{V}}_{j}$ , where $\hat{\theta }=\hat{E}\left(h(X)\right)$ , and the empirical of mean for h(X) and $V_{j}$ can be calculated by the aggregated data shown in Supplementary Materials.

With the different forms of $w_{j}$ , the CMA is designed to target different causal estimands tailored to specific populations. In the following subsections, we define the target population for each target estimand—ATE, ATT, ATC, and ATO. Subsequently, we present the corresponding forms of $w_{j}$ for CMA in the context of ATE, ATT, ATC, and ATO. In addition, we establish the relationship between CMA and traditional meta-analysis. Finally, we present the connections between ATE, ATT, ATC, and ATO.

### 2.3 ATE, inverse probability weighting, and weighting by study size

The ATE is E(Y(1)−Y(0)) , which is equal to WATE if h(X)=1 . The target population of ATE is interpreted as the patients from both the treated and control groups across all studies. Under such case, $m_{ij}$ in the control arm is $\frac{1}{1-{\hat{e}}_{ij}}$ and $m_{ij}$ in the treatment arm is $\frac{1}{{\hat{e}}_{ij}}$ , also known as the inverse probability weighting. With Equation (3) under the constant of the function, h=1 , the proposed CMA estimator will be in the following formula:

(4) $$\begin{array}{r} \sum_{j=1}^{K}\frac{{\hat{p}}_{1j}n_{j}}{n}-\sum_{j=1}^{K}\frac{{\hat{p}}_{0j}n_{j}}{n}.\; \end{array}$$

It has the causal interpretation, that is, the estimated pooled event rate for the treatment arm versus the control arm, whereas the subjects counterfactually from both comparison groups are given the treatment or control. Interestingly, we find that it is equivalent to the traditional meta-analysis estimator weighted by the study-specific sample sizes, $w_{j}^{ATE}=\frac{n_{j}}{n}$ . The derived closed-form variance for CMA-targeted ATE is

$$\begin{array}{rl} V^{ATE} & =n^{-2}\sum_{j=1}^{K}n_{1j}{\hat{p}}_{1j}{\left[e_{j}^{-1}\left(1-{\hat{\tau }}_{1}\right)+\left(1-e_{j}\right)M_{j}\right]}^{2}+n_{1j}\left(1-{\hat{p}}_{1j}\right){\left[e_{j}^{-1}{\hat{\tau }}_{1}+\left(1-e_{j}\right)M_{j}\right]}^{2}\\ & \;+n_{0j}{\hat{p}}_{0j}{\left[{\left(1-e_{j}\right)}^{-1}\left(1-{\hat{\tau }}_{0}\right)+e_{j}M_{j}\right]}^{2}+n_{0j}\left(1-{\hat{p}}_{0j}\right){\left[{\left(1-e_{j}\right)}^{-1}{\hat{\tau }}_{0}-e_{j}M_{j}\right]}^{2}, \end{array}$$

where $M_{j}=-e_{j}^{-1}\left({\hat{p}}_{1j}-{\hat{\tau }}_{1}\right)-{\left(1-e_{j}\right)}^{-1}\left({\hat{p}}_{0j}-{\hat{\tau }}_{0}\right)$ , ${\hat{\tau }}_{1}=\sum_{j=1}^{K}\frac{{\hat{p}}_{1j}n_{j}}{n}$ , and ${\hat{\tau }}_{0}=\sum_{j=1}^{K}\frac{{\hat{p}}_{0j}n_{j}}{n}$ .

### 2.4 ATT and ATC

If our target estimand is ATT, that is, $\tau_{w}=E\left(Y(1)-Y(0)|A=1\right)$ with h(X)=e(X) . The target population for ATT is the patients from the treated groups across all studies. Under such case, when substituting h(X)=e(X) into Equation (3), the formula of the CMA estimator will be as follows:

(5) $$\begin{array}{r} \sum_{j=1}^{K}\frac{n_{1j}}{n_{T}}\left({\hat{p}}_{1j}-{\hat{p}}_{0j}\right),\; \end{array}$$

where $n_{T}=\sum_{j=1}^{K}n_{1j}$ denotes the number of subjects in the treated groups across studies. The weights are $w_{j}^{ATT}=\frac{n_{1j}}{n_{T}}$ . The variance of CMA for ATT is

$$\begin{array}{rl} V^{ATT} & =n_{T}^{-2}\sum_{j=1}^{K}n_{1j}{\hat{p}}_{1j}{\left[\left(1-{\hat{\tau }}_{1}\right)+\left(1-e_{j}\right)M_{j}\right]}^{2}+n_{1j}\left(1-{\hat{p}}_{1j}\right){\left[-{\hat{\tau }}_{1}+\left(1-e_{j}\right)M_{j}\right]}^{2}\\ & \;+n_{0j}{\hat{p}}_{0j}{\left[{\left(1-e_{j}\right)}^{-1}\left(1-{\hat{\tau }}_{0}\right)e_{j}+e_{j}M_{j}\right]}^{2}+n_{0j}\left(1-{\hat{p}}_{0j}\right){\left[{\left(1-e_{j}\right)}^{-1}{\hat{\tau }}_{0}e_{j}-e_{j}M_{j}\right]}^{2}, \end{array}$$

where the $M_{j}=-{\left(1-e_{j}\right)}^{-1}\left({\hat{p}}_{0j}-{\hat{\tau }}_{0}\right)$ , ${\hat{\tau }}_{1}=\sum_{j=1}^{K}\frac{n_{1j}}{n_{T}}\;{\hat{p}}_{1j}$ , and ${\hat{\tau }}_{0}=\sum_{j=1}^{K}\frac{n_{1j}}{n_{T}}\;{\hat{p}}_{0j}$ .

Similarly, if our target estimand is ATC, that is, $\tau_{w}=E\left(Y(1)-Y(0)|A=0\right)$ with h(X)=1−e(X) . The target population of ATC is the patients from the control groups across all studies. Under such case, the proposed CMA estimator is

(6) $$\begin{array}{r} \sum_{j=1}^{K}\frac{n_{0j}}{n_{C}}\left({\hat{p}}_{1j}-{\hat{p}}_{0j}\right),\; \end{array}$$

where $n_{C}=\sum_{j=1}^{K}n_{0j}$ denotes the number of subjects in the control across studies. The weights are $w_{j}^{ATC}=\frac{n_{0j}}{n_{C}}$ . The variance of CMA for ATC is

$$\begin{array}{rl} V^{ATC} & =n_{C}^{-2}\sum_{j=1}^{K}n_{1j}{\hat{p}}_{1j}{\left[e_{j}^{-1}\left(1-{\hat{\tau }}_{1}\right)\left(1-e_{j}\right)+\left(1-e_{j}\right)M_{j}\right]}^{2}\\ & \;+n_{1j}\left(1-{\hat{p}}_{1j}\right){\left[-e_{j}^{-1}{\hat{\tau }}_{1}\left(1-e_{j}\right)+\left(1-e_{j}\right)M_{j}\right]}^{2}+n_{0j}{\hat{p}}_{0j}{\left[-\left(1-{\hat{\tau }}_{0}\right)-e_{j}M_{j}\right]}^{2}\\ & \;+n_{0j}\left(1-{\hat{p}}_{0j}\right){\left[{\hat{\tau }}_{0}-e_{j}M_{j}\right]}^{2}, \end{array}$$

where the $M_{j}=-e_{j}^{-1}\left({\hat{p}}_{1j}-{\hat{\tau }}_{1}\right),{\hat{\tau }}_{1}=\sum_{j=1}^{K}\frac{n_{0j}}{n_{C}}\;{\hat{p}}_{1j},$ and ${\hat{\tau }}_{0}=\sum_{j=1}^{K}\frac{n_{0j}}{n_{C}}\;{\hat{p}}_{0j}$ .

### 2.5 ATO, overlap weighting, and Mantel–Haenszel estimators

For the overlap weighting, the function for the targeted overlap population is h(X)=e(X)(1−e(X)) . Thus, we used the $m_{ij}=1-{\hat{e}}_{ij}$ if the i th subject is in the treated arm and $m_{ij}={\hat{e}}_{ij}$ for the i th subject in the control arm. The target population of ATO is the population of patients who exhibit the greatest clinical equipoise or highest uncertainty regarding both comparison groups. By using the overlap weights correspondingly, the proposed CMA estimator is reduced to

(7) $$\begin{array}{r} {\left(\sum_{j=1}^{K}\frac{n_{0j}n_{1j}}{n_{0j}+n_{1j}}\right)}^{-1}\sum_{j=1}^{K}\frac{n_{0j}n_{1j}}{n_{0j}+n_{1j}}\left({\hat{p}}_{1j}-{\hat{p}}_{0j}\right).\; \end{array}$$

From Equation (5), it can be seen as the weighted average estimator with the weight being the Mantel–Haenszel weights. Thus, the target estimand of the Mantel–Haenszel estimators is the ATO. The weights are $w_{j}^{ATO}={\left(\sum_{j=1}^{K}\frac{n_{0j}n_{1j}}{n_{0j}+n_{1j}}\right)}^{-1}\frac{n_{0j}n_{1j}}{n_{0j}+n_{1j}}$ . The variance of CMA under ATO is

$$\begin{array}{rl} V^{ATO} & ={\left(\sum_{j=1}^{K}\frac{n_{0j}n_{1j}}{n_{0j}+n_{1j}}\right)}^{-2}\sum_{j=1}^{K}n_{1j}{\hat{p}}_{1j}{\left(1-e_{j}\right)}^{2}{\left[\left(1-{\hat{\tau }}_{1}\right)+M_{j}\right]}^{2}\\ & \;+n_{1j}\left(1-{\hat{p}}_{1j}\right){\left(1-e_{j}\right)}^{2}{\left[-{\hat{\tau }}_{1}+M_{j}\right]}^{2}+n_{0j}{\hat{p}}_{0j}e_{j}^{2}{\left[\left(1-{\hat{\tau }}_{0}\right)+M_{j}\right]}^{2}\\ & \;+n_{0j}\left(1-{\hat{p}}_{0j}\right)e_{j}^{2}{\left[{\hat{\tau }}_{0}-M_{j}\right]}^{2}, \end{array}$$

where $M_{j}=-e_{j}\left({\hat{p}}_{1j}-{\hat{\tau }}_{1}\right)-\left(1-e_{j}\right)\left({\hat{p}}_{0j}-{\hat{\tau }}_{0}\right),{\hat{\tau }}_{1}={\left(\sum_{j=1}^{K}\frac{n_{0j}n_{1j}}{n_{0j}+n_{1j}}\right)}^{-1}\sum_{j=1}^{K}\frac{n_{0j}n_{1j}}{n_{0j}+n_{1j}}{\hat{p}}_{1j},$ and ${\hat{\tau }}_{0}={\left(\sum_{j=1}^{K}\frac{n_{0j}n_{1j}}{n_{0j}+n_{1j}}\right)}^{-1}\sum_{j=1}^{K}\frac{n_{0j}n_{1j}}{n_{0j}+n_{1j}}{\hat{p}}_{0j}$ . The target population size can be different for different target estimands when the randomized ratios are different across studies, and an example of target estimands and population sizes is illustrated in Supplementary Appendix S1.

### 2.6 Connection between ATE, ATT, ATC, and ATO

When the allocation ratios across all studies are consistent, such that for the j th study ( j=1,…,K ), the ratio $\frac{n_{0j}}{n_{1j}}=c$ , where c is a constant, we find ourselves in an ideal scenario where treatment A and study indicator X are uncorrelated. In this case, the weights derived from CMA for ATE, ATT, ATC, and ATO are shown in Equations (4) to (7) and are identical. As detailed in the Supplementary Material, the weights from CMA are uniformly $\frac{n_{j}}{\sum_{j=1}^{K}n_{j}}$ . Consequently, the estimates for ATE, ATT, ATC, and ATO are identical when the randomization ratios across studies are identical. It is worth noting that the sample size of the different target populations is not assumed to be the same under such a scenario.

## 3 Results

To validate our proposed CMA estimators, we conducted an analysis on the real-world data using target estimand ATE, ATT, ATC, and ATO. The motivating study by Chou et al.^37^ investigated the association between statin use and all-cause mortality. Across the five included trials, the mean age of participants ranged from 57 to 62 years. Treatment was defined as statin therapy, and the control arm consisted of placebo recipients. The primary outcome was all-cause mortality occurring within the follow-up period, which varied between 1 and 3 years across studies.

The aggregated data are presented in Table 1, where we report the number of subjects in both the treated and control arms, along with the events for each group. Additionally, we conducted the CMA for the target estimands—ATT, ATE, ATC, and ATO—and displayed the corresponding study weights based on each estimand.

**Table 1:** Aggregated data from five studies for the intervention of statin versus placebo control, such as the number of subjects within the treated or control arm and the events for each arm. In addition, we showed the specified weights from CMA for the target estimands ATE, ATT, ATC, and ATO in the last four columns Table 1 Long description.

			Event	Event				
	Number	Number	number	number				
	of	of	within	within				
	subjects in	subjects in	each study	each study				
	the	the	in the	in the	Weights	Weights	Weights	Weights
	treatment	control	treatment	control	for	for	for	for
Study	arm	arm	arm (%)	arm (%)	ATE	ATT	ATC	ATO
ACPCS^38^	460	459	1	8	0.30	0.23	0.40	0.34
Beishuizen^39^	103	79	3	4	0.06	0.05	0.07	0.07
Bone^40^	485	119	0	0	0.19	0.25	0.10	0.14
KAPS^41^	214	212	3	4	0.14	0.11	0.18	0.16
METEOR^42^	700	281	1	0	0.32	0.36	0.24	0.30

**Figure 2:**
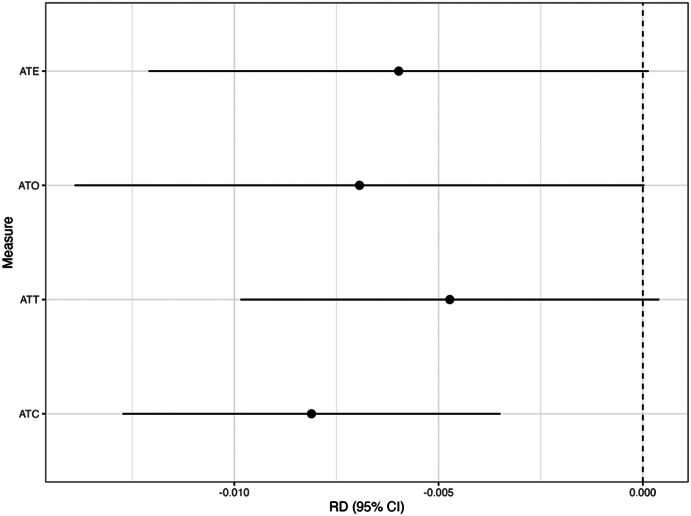
Results for the CMA estimator. We show the estimate with the 95% confidence interval (CI) for the target estimand of ATE, ATT, ATC, and ATO in the risk difference (RD) scale with the CMA estimator.

Figure 2 shows the results for the CMA estimators with the target estimands of ATE, ATT, ATC, and ATO. From Figure 2, we found that using the CMA estimator can vary depending on the choice of the target estimands. For example, suppose the target estimand is ATT, but we instead use the CMA estimator for the ATC. The ATC estimate is statistically significant (risk difference [RD]: –0.008; 95% confidence interval [CI]: –0.012 to –0.003), whereas the CMA estimator for the ATT yields a nonsignificant result (RD: –0.005; 95% CI: –0.010 to 0.001). This difference in inference indicates that conclusions can change meaningfully depending on the target population. Therefore, we recommend prespecifying the target population before conducting the treatment evaluation.

## 4 Discussion

In this study, we introduce the CMA estimator, specifically designed for the WATE, which utilizes aggregated data to effectively address between-study confounding effects arising from heterogeneous sample size ratios. Estimators corresponding to ATE, ATT, ATC, and ATO are provided. Additionally, we establish the relationship between the CMA for various causal estimands and traditional fixed-effect meta-analysis estimators such as those weighted by sample size and Mantel–Haenszel weights. Through a concrete meta-analysis example in real-world data, we illustrate the distinctions between these target estimands and their corresponding estimators.

One of the advantages of the CMA framework is its ability to rely solely on aggregated data rather than IPD. Given that access to IPD is often restricted due to privacy concerns or logistical limitations for access to the clinical trials, our method provides a practical alternative for researchers who need causal estimates based on available summary-level statistics for clinical trials.

Additionally, the CMA framework addresses between-study confounding effects. Unequal randomization ratios between treated and untreated groups across studies can lead to biased estimates if not properly adjusted. By applying propensity score weighting to create pseudo-populations where confounders are balanced, the CMA framework effectively mitigates these biases. This approach not only aligns with causal inference methods but also enhances the generalizability of the results to different target populations.

We demonstrated that under unequal randomization ratios, the proposed CMA framework is equivalent to traditional meta-analysis methods. For example, in the case of ATO, the CMA weights are identical to the Mantel–Haenszel weights, and for ATE, the CMA weights correspond to the study-specific sample size weights used in traditional meta-analyses. This equivalence highlights how traditional meta-analysis can provide a clear causal interpretation of treatment effects under unequal randomized ratios, effectively bridging the gap between conventional meta-analytic techniques and causal inference methodologies.

Unlike previous work on causally interpretable meta-analysis using IPD,^20^^–^ ^23^ which often defines the target populations through the distribution of effect modifiers or external covariates, our approach defines the target population as the combined population of the included studies. This design-based perspective is consistent with Higgins et al.^43^ and Rice et al.^44^ and avoids assumptions that studies are sampled from a broader super-population. By grounding inference in the actual populations studied, this approach facilitates a more clinically meaningful interpretation of the ATE and other related estimands in the presence of heterogeneous treatment effects.

Our study has some limitations. It is important to note that our focus is currently on the fixed-effect meta-analysis setting. If the treatment effects across studies are heterogeneous and are assumed to follow a specific distribution, such as a normal distribution (which is often unjustified),^45^ we plan to address this issue by developing a new causal framework for random-effect meta-analysis estimators. Details of this framework will be reported in future work.

A common concern when synthesizing evidence from heterogeneous trials is the potential violation of the consistency assumption due to variation in treatment versions. In our real-world application, we used the set of trials reported by Chou et al.,^37^ where the treatment group was consistently defined as statin use and the control group as placebo. Therefore, we consider the consistency assumption to be reasonably satisfied in this context. In future work, we plan to investigate causal meta-analytic methods that explicitly account for variation in treatment versions in settings where consistency may not be applicable.

While a key contribution of this work is that the proposed methods require only aggregate data, it is important to acknowledge the inherent limitations relative to methods based on IPD.^20^^–^ ^23^ In particular, IPD-based approaches allow for more flexible modeling of between-study heterogeneity and enable the definition of target populations based on the distribution of arbitrary effect modifiers, which is not directly feasible using only aggregate data.

Our framework addresses between-study confounding related to design features such as the baseline risks. However, it does not fully adjust for covariates that differ across studies and modify treatment effects. An extension using meta-regression with aggregate covariates could potentially address such heterogeneity.

We assume that, within each study, confounding variables are absent due to the randomization procedure. While this assumption is reasonable for randomized controlled trials (RCTs), it may not hold in observational studies or real-world data, where additional confounders may exist. In such cases, the causal interpretation of the CMA and its target populations may be compromised. Further research on causally interpretable meta-analysis for observational studies, using only aggregated data, will be valuable. In addition, while our framework is presented for binary outcomes, the core ideas readily extend to continuous outcomes, and future work may explore more complex outcome types, such as time-to-event data, with appropriate methodological adaptations.

In many real-world scenarios, IPD may be available for only a subset of relevant trials, while others are accessible only through published aggregate results. Our current framework is designed to work with aggregated data (AD) alone, ensuring broad applicability even when IPD is not accessible. However, when IPD is available for some studies, it presents an opportunity to enhance estimation through covariate adjustment and richer modeling of effect heterogeneity. Recent work^46^ has proposed hybrid approaches that borrow information from IPD to augment the estimates from AD in a unified meta-analytic framework. Extending our approach to such partially observed IPD settings is a promising direction for future research, potentially improving both efficiency and causal interpretability.

## Supporting information

10.1017/rsm.2025.10069.sm001 Zhang et al. supplementary material Zhang et al. supplementary material

## Data Availability

Data are provided in Table 1.

## Supplementary material

To view supplementary material for this article, please visit http://doi.org/10.1017/rsm.2025.10069.

## Long descriptions

### Table 1 Long description

The table consists of nine columns and six rows including the header.

Column headers from left to right are: Study, Number of subjects in the treatment arm, Number of subjects in the control arm, Event number within each study in the treatment arm (percent), Event number within each study in the control arm (percent), Weights for A T E, Weights for A T T, Weights for A T C, and Weights for A T O.

Data rows:

* A C P C S: 460 treatment, 459 control; Events: 1 treatment, 8 control; Weights: A T E 0.30, A T T 0.23, A T C 0.40, A T O 0.34.

* Beishuizen: 103 treatment, 79 control; Events: 3 treatment, 4 control; Weights: A T E 0.06, A T T 0.05, A T C 0.07, A T O 0.07.

* Bone: 485 treatment, 119 control; Events: 0 treatment, 0 control; Weights: A T E 0.19, A T T 0.25, A T C 0.10, A T O 0.14.

* K A P S: 214 treatment, 212 control; Events: 3 treatment, 4 control; Weights: A T E 0.14, A T T 0.11, A T C 0.18, A T O 0.16.

* M E T E O R: 700 treatment, 281 control; Events: 1 treatment, 0 control; Weights: A T E 0.32, A T T 0.36, A T C 0.24, A T O 0.30.

Navigate back to Table 1.
