# Supplementary material for: A causal meta-analysis framework for clinical trials with unequal randomization ratios
Source: Res Synth Methods. 2026 Mar 5;17(4):770–81. doi: 10.1017/rsm.2025.10069 (PMC13311351; doi:10.1017/rsm.2025.10069)
Supplement: Zhang et al. supplementary material [file S1759287925100690sup001.docx]

Appendix to “A Causal Meta-Analysis Framework for Clinical Trials with Unequal Randomization Ratios”

Table of Contents

[Section S1. Different target population sizes for different causal estimands 2](#_Toc178668414)

[Section S2. The derivation of the CMA estimator 5](#_Toc178668415)

[Section S3. The derivation of the variance of the CMA estimator 6](#_Toc178668416)

# Section S1. Different target population sizes for different causal estimands

To demonstrate how the population sizes for the causal estimands vary, we created a 2x2 table using an example involving a total of 400 patients across two studies. Specifically, the number within the $j$th row ($j=1,2$) and the $a$th column ($a=1, 2$) is the number of patients within the $x$th study for the treated ($a=1$) or control group ($a=2$). Detailed patient counts are shown in Table S1.

| Study | $\boldsymbol{n}_{\mathbf{1}\boldsymbol{j}}$ | $\boldsymbol{n}_{\mathbf{0}\boldsymbol{j}}$ |
| --- | --- | --- |
| 1 | 100 | 100 |
| 2 | 40 | 160 |

Table S1. An example for studies.

ATE. The target population of ATE is interpreted as the patients from both the treated and control groups across all studies. In our specific example, the total population size is 400, with half deriving from study 1 and the other half from Study 2 as shown in Table S2.

ATT. The target population for ATT is the patients from the treated groups across all studies. For instance, in our analysis, the total number comprises 140 patients, with 100 from the treated group in Study 1 and 40 from the treated group in Study 2.

ATC. The target population of ATC is the patients from the control groups across all studies. In our example, the total number is 260 where 100 patients are from the control arm in the Study 1 and 40 patients are from the control arm in the Study 2.

ATO. The target population of ATO is the population of patients who exhibit the greatest clinical equipoise or highest uncertainty regarding both comparison groups. The population size is less than the number of patients in the treated and control arm from all studies. The total number is 82, representing the smallest group among the study populations. It includes 50 patients from Study 1 and 32 patients from Study 2. The definition of the target population arises from the patients with the highest level of uncertainty, allowing for two ways to identify these patients:

(a) The proportion of these patients within the treated arm should equal the proportion remaining the control arm after these patients are excluded from the control arm.

(b) The proportion of patients remaining in the treatment arm after excluding these patients should equal their proportion in the control arm.

Using the description from (a) or (b), we calculate the number of patients for the overlap population. For example, in the Study 2, with y representing the number of patients in the overlap population. This gives us

$\frac{y}{40}=\frac{160-y}{160}$ or $\frac{40-y}{40}=\frac{y}{160}$

Both ways are equivalent to calculate $y=\frac{n_{0j}n_{1j}}{n_{0j}+n_{1j}}$ with $n_{0j}=40$ and $n_{1j}=160$, which yield the answer with $y=32$. This confirms the size of the overlap population from the Study 2.

|  | Weights for each study from different target population | | | |
| --- | --- | --- | --- | --- |
|  | after weighting by CMA for ATE | after weighting by CMA for ATT | after weighting by CMA for ATC | after weighting by CMA for ATO |
| Study |  |  |  |  |
| 1 | 0.5 | 0.71 | 0.38 | 0.61 |
| 2 | 0.5 | 0.29 | 0.62 | 0.39 |

Table S2. An example for number of target population for different target estimands.

# Section S2. The derivation of the CMA estimator

From the estimator for the WATE specified in the Equation (1) in the main manuscript, we have

$$\hat{\tau}_{w}=\frac{\sum_{j=1}^{K} \sum_{i=1}^{n_{j}} A_{ij}Y_{ij}\frac{\hat{h}\left( e_{ij} \right)}{\hat{e}_{ij}}}{\sum_{j=1}^{K} \sum_{i=1}^{n_{j}} A_{ij}\frac{\hat{h}\left( e_{ij} \right)}{\hat{e}_{ij}}}- \frac{\sum_{j=1}^{K} \sum_{i=1}^{n_{j}} \left( 1-A_{ij} \right)Y_{ij}\frac{\hat{h}\left( X_{ij} \right)}{1-\hat{e}_{ij}}}{\sum_{j=1}^{K} \sum_{i=1}^{n_{j}} \left( 1-A_{ij} \right)\frac{\hat{h}\left( X_{ij} \right)}{1-\hat{e}_{ij}}}.$$

As we illustrated the $\hat{e}_{ij}$ as the propensity score and as a function of the study indicator, we have for the $j$th study, $\hat{e}_{ij}=\hat{\lambda}_{j}$. This suggests that the participants within the $j$th study have the same value of $\hat{e}_{ij}$, which gives us

$$\sum_{j=1}^{K} \sum_{i=1}^{n_{j}} A_{ij}Y_{ij}\frac{\hat{h}\left( e_{ij} \right)}{\hat{e}_{ij}}=\sum_{j=1}^{K} \frac{n_{1j}\hat{p}_{1j}\hat{h}_{j}}{\hat{\lambda}_{j}}=\sum_{j=1}^{K} n_{j}\hat{h}_{j}\hat{p}_{1j},$$

$$\sum_{j=1}^{K} \sum_{i=1}^{n_{j}} A_{ij}\frac{\hat{h}\left( e_{ij} \right)}{\hat{e}_{ij}}=\sum_{j=1}^{K} \frac{n_{1j}\hat{h}_{j}}{\hat{\lambda}_{j}}=\sum_{j=1}^{K} n_{j}\hat{h}_{j},$$

$$\sum_{j=1}^{K} \sum_{i=1}^{n_{j}} \left( 1-A_{ij} \right)Y_{ij}\frac{\hat{h}\left( X_{ij} \right)}{1-\hat{e}_{ij}}=\sum_{j=1}^{K} \frac{n_{0j}\hat{p}_{0j}\hat{h}_{j}}{{1-\hat{\lambda}}_{j}}=\sum_{j=1}^{K} n_{j}\hat{h}_{j}\hat{p}_{0j},$$

$$\sum_{j=1}^{K} \sum_{i=1}^{n_{j}} \left( 1-A_{ij} \right)\frac{\hat{h}\left( X_{ij} \right)}{1-\hat{e}_{ij}}=\sum_{j=1}^{K} \frac{n_{0j}\hat{h}_{j}}{{1-\hat{\lambda}}_{j}}=\sum_{j=1}^{K} n_{j}\hat{h}_{j}.$$

This gives us the formula for the CMA estimator.

# Section S3. The derivation of the variance of the CMA estimator

An estimator for the propensity score can be obtained by fitting a logistic regression model, $P(Y_{ij}|X_{ij},\beta)$ indexed by $\beta=(\beta_{1},\ldots,\beta_{K})$, a vector of coefficients of the study indicator with no intercept term. The maximum likelihood estimate of the logistic regression gives a closed form for the estimator of $\beta_{j}$ for the $j$th study if we assume there is no intercept term and only study indicator ${I(X}_{ij}=j)$ $j=1,\ldots,K$ in the model, that is,

$$\hat{\beta}_{j}=\log\left( \frac{n_{1j}}{n_{0j}} \right). (S1)$$

From the equation (S1), we have the estimator of the propensity score, which is

$$\hat{\lambda}_{j}=\hat{\Pr} (A_{ij}=1|X_{ij})=\frac{n_{1j}}{n_{j}}. (S2)$$

We can write down the influence function of $\beta$, that is,

$$\sqrt{n}\left( \hat{\beta}-\beta\right)=E_{\beta\beta}^{-1}\frac{1}{\sqrt{n}}\sum_{j=1}^{K} \sum_{i=1}^{n_{j}} \left( A_{ij}-e_{ij} \right)X_{ij}. (S3)$$

It can be found by the aggregated data from the following equation,

$$\sqrt{n}\left( \hat{\beta}-\beta\right)=E_{\beta\beta}^{-1}\frac{1}{\sqrt{n}}\sum_{j=1}^{K} n_{1j}-n_{j}e_{j} (S4)$$

where $E_{\beta\beta}$ is a diagonal matrix with K dimension and the $j$th diagonal element is $n_{j}\frac{e_{j}\left( 1-e_{j} \right)}{n}$.

The variance of the $\hat{\tau}_{CMA}$ can be calculated by finding the influence function of $\tau_{w}$. We restrict the $h\left( X \right)=h\left( e\left( X \right) \right)$, that is $h\left( X \right)$ is a function of the propensity score function, which is general assumption as the target estimands, ATE, ATT, ATC, and ATO are using the $h\left( e\left( X \right) \right)$ to be 1, $e\left( X \right)$, $1-e\left( X \right)$, and $e\left( X \right)\left( 1-e\left( X \right) \right).$ Then, we write down the influence function of $\tau_{w}$ by individual-level data, which is,

$$\sqrt{n}\left( \hat{\tau}_{CMA}-\tau_{w} \right)=\theta^{-1}\frac{1}{\sqrt{n}} \sum_{j=1}^{K} \sum_{i=1}^{n_{j}} I_{ij}, (S5)$$

where

$I_{ij}=\frac{A_{ij}\left( Y_{ij}-\tau_{1} \right)h_{ij}}{e_{ij}}-\frac{\left( 1-A_{ij} \right)\left( Y_{ij}-\tau_{0} \right)h_{ij}}{1-e_{ij}}+\left( A_{ij}-e_{ij} \right)H_{\beta}^{T}E_{\beta\beta}^{-1}$,

$$H_{\beta}=\frac{1}{n}\sum_{j=1}^{K} \sum_{i=1}^{n_{j}} \frac{A_{ij}\left( Y_{ij}-\tau_{1} \right)\left( 1-e_{ij} \right)\left( h_{ij}^{'}e_{ij}-h_{ij} \right)X_{ij}}{e_{ij}}-\frac{\left( 1-A_{ij} \right)\left( Y_{ij}-\tau_{0} \right)e_{ij}\left( h_{ij}^{'}(1-e_{ij})+h_{ij} \right)X_{ij}}{{1-e}_{ij}}$$

, and

$\theta=E\left( h(X) \right)$.

To see the influence function can be obtained by the aggregated data, we first notice that the jth element of the vector $H_{\beta}$ can be written as

$$n_{1j}e_{j}^{-1}\left( \hat{p}_{1j}-\hat{\tau}_{1} \right)\left( h_{j}^{'}e_{j}-h_{j} \right)\left( 1-e_{j} \right)-n_{0j}\left( 1-e_{j} \right)^{-1}\left( \hat{p}_{0j}-\hat{\tau}_{0} \right)\left( h_{j}^{'}\left( 1-e_{j} \right)+h_{j} \right)e_{j},$$

where $h_{j}^{'}$ is derivative of $h(e_{j})$ at $e_{j}$.

So that we have the jth element of $H_{\beta}^{T}E_{\beta\beta}^{-1}$ is

$$M_{j}=\frac{n_{1j}e_{j}^{-1}\left( \hat{p}_{1j}-\hat{\tau}_{1} \right)\left( h_{j}^{'}e_{j}-h_{j} \right)\left( 1-e_{j} \right)-n_{0j}\left( 1-e_{j} \right)^{-1}\left( \hat{p}_{0j}-\hat{\tau}_{0} \right)\left( h_{j}^{'}\left( 1-e_{j} \right)+h_{j} \right)e_{j}}{n_{j}e_{j}\left( 1-e_{j} \right)}.$$

Furthermore, $\sum_{j=1}^{K} \sum_{i=1}^{n_{j}} I_{ij}$ can be rewritten by the aggregated data by noticing that

$$\sum_{j=1}^{K} \sum_{i=1}^{n_{j}} I_{ij}=\sum_{j=1}^{K} \sum_{i=1}^{n_{j}} \sum_{a=0}^{1} \sum_{y=0}^{1} I\left( A_{ij}=a \right)I\left( Y_{ij}=y \right)I\left( X_{ij}=j \right)B_{ayj}$$

where $B_{00j}=\left( 1-e_{j} \right)^{-1}\hat{\tau}_{0}h_{j}-e_{j}M_{j}$, $B_{01j}=-\left( 1-e_{j} \right)^{-1}(1-\hat{\tau}_{0})h_{j}-e_{j}M_{j}$, $B_{10j}=-e_{j}^{-1}\hat{\tau}_{1}h_{j}+\left( 1-e_{j} \right)M_{j}$, and $B_{11j}=e_{j}^{-1}\left( 1-\hat{\tau}_{1} \right)h_{j}+\left( 1-e_{j} \right)M_{j}$.

Combining the results from the above, we will have that

$$\sqrt{n}\left( \hat{\tau}_{CMA}-\tau_{w} \right)= \theta^{-1}\frac{1}{\sqrt{n}}\sum_{j=1}^{K} n_{1j}\hat{p}_{1j}B_{11j}+n_{1j}\left( 1-\hat{p}_{1j} \right)B_{10j}+n_{0j} \hat{p}_{0j}B_{01j}+n_{0j}\left( 1-\hat{p}_{0j} \right)B_{00j}.$$

The variance estimator of the  $\hat{\tau}_{CMA}$ can thus be obtained by

$$\left( n^{2} \hat{\theta}^{2} \right)^{-1}\sum_{j=1}^{K} \hat{V}_{j}$$

where the $\hat{V}_{j}=n_{1j}\hat{p}_{1j}\hat{B}_{11j}^{2}+n_{1j}\left( 1-\hat{p}_{1j} \right)\hat{B}_{10j}^{2}+n_{0j}\hat{p}_{0j}\hat{B}_{01j}^{2}+n_{0j}\left( 1-\hat{p}_{0j} \right)\hat{B}_{00j}^{2}$ and $\hat{\theta}=\frac{1}{n}\sum_{j=1}^{K} n_{j}\hat{h}_{j}$.

We can apply the equations for the variance of the CMA estimator under the ATE, ATO, ATT, and ATC and found their corresponding variances.

**Connection between ATE, ATT, ATC, and ATO**

If we assume that for $j$th study ($j=1,\ldots,K$), the ratio $\frac{n_{0j}}{n_{1j}}=c$, where $c$ is a constant, we have the same results from CMA for ATE, ATT, ATC, and ATO. This is because

$$\frac{n_{1j}}{\sum_{j=1}^{K} n_{1j}}=\frac{(c+1)n_{1j}}{(c+1)\sum_{j=1}^{K} n_{1j}}=\frac{n_{1j}+cn_{1j}}{\sum_{j=1}^{K} n_{1j}+cn_{1j}}=\frac{n_{j}}{\sum_{j=1}^{K} n_{j}} (S6)$$

$$\frac{n_{0j}}{\sum_{j=1}^{K} n_{0j}}=\frac{cn_{1j}}{\sum_{j=1}^{K} cn_{1j}}=\frac{{cn}_{1j}}{c\sum_{j=1}^{K} n_{1j}}=\frac{n_{1j}}{\sum_{j=1}^{K} n_{1j}}$$

Then, substitute equation S6,

$$\frac{n_{0j}}{\sum_{j=1}^{K} n_{0j}}=\frac{n_{j}}{\sum_{j=1}^{K} n_{j}}$$

Similarly,

$$\left( \sum_{j=1}^{K} \frac{n_{1j}n_{0j}}{n_{1j}+n_{0j}} \right)^{-1}\frac{n_{1j}n_{0j}}{n_{1j}+n_{0j}}=\left( \sum_{x=1}^{K} \frac{c^{2}n_{1j}}{(c+1)n_{1j}} \right)^{-1}\frac{c^{2}n_{1j}}{(c+1)n_{1j}}=\frac{{\frac{c^{2}}{c+1}n}_{1j}}{\frac{c^{2}}{c+1}\sum_{j=1}^{K} n_{1j}}=\frac{n_{1j}}{\sum_{j=1}^{K} n_{1j}}$$

Then, substitute Equation S6,

$$\left( \sum_{j=1}^{K} \frac{n_{1j}n_{0j}}{n_{1j}+n_{0j}} \right)^{-1}\frac{n_{1j}n_{0j}}{n_{1j}+n_{0j}}=\frac{n_{j}}{\sum_{j=1}^{K} n_{j}}$$
